# Coarsening behavior of bulk nanobubbles in water

**DOI:** 10.1038/s41598-021-98783-2

**Published:** 2021-09-27

**Authors:** Jeong Il Lee, Han Sol Huh, Joong Yull Park, Jung-Geun Han, Jong-Min Kim

**Affiliations:** 1 School of Mechanical Engineering, Chung-Ang University, Seoul, 06974 Republic of Korea; 2 School of Civil and Environmental Engineering, Chung-Ang University, Seoul, 06974 Republic of Korea; 3 Department of Intelligent Energy and Industry, Chung-Ang University, Seoul, 06974 Republic of Korea

**Keywords:** Synthesis and processing, Experimental particle physics

## Abstract

In recent years, minuscule gas bubbles called bulk nanobubbles (BNBs) have drawn increasing attention due to their unique properties and broad applicability in various technological fields, such as biomedical engineering, water treatment, and nanomaterials. However, questions remain regarding the stability and behavior of BNBs. In the present work, BNBs were generated in water using a gas–liquid mixing method. NB analysis was performed using a nanoparticle tracking analysis (NTA) method to investigate the coarsening behavior of BNBs in water over time. The diameters of the BNBs increased, and their cubic radii increased linearly (r^3^ ~ t) over time. While the concentration of BNBs decreased, the total volume of BNBs remained the same. The size distribution of the BNBs broadened, and the concentration of larger BNBs increased over time. These results indicate that relatively small BNBs disappeared due to dissolution and larger BNBs grew through mass transfer between BNBs instead of coalescence. In other words, BNBs underwent Ostwald ripening: gas molecules from smaller BNBs diffused through the continuous phase to be absorbed into larger BNBs.

## Introduction

In recent years, researchers have increasingly investigated bulk nanobubbles (BNBs) due to their unique characteristics, such as long lifespan^1^, metabolism acceleration^2^, promotion of fossil fuel combustion^3,4^, cleaning capabilities^5,6^, and utility as both a contrast and drug delivery agent^7^. However, questions remain regarding the stability of BNBs and the principles of their application. According to the Young–Laplace equation, the internal pressure within BNBs is much greater than atmospheric pressure. Consequently, BNBs are unstable under atmospheric conditions. Ohgaki et al.^8^ reported that the internal pressure of BNBs is about twice as large as calculated using the Young–Laplace equation. They generated BNBs from three different gases (N_2_, CH_4_, and Ar) and showed that their internal pressures were 5.6–6.3 MPa with a 50 nm radius for each NB; the corresponding internal pressure predicted by the Young–Laplace equation was about 2.9 MPa. In contrast, the internal pressure of the BNBs has also been reported to be lower than that calculated using the Young–Laplace equation. Nagayama et al.^9^ used molecular dynamics simulation to show that there are few vapor atoms in nanobubbles. They concluded that the internal pressure of a nanobubble is much lower than that estimated by the Young–Laplace equation because nanobubbles have few atoms.

BNBs are potential materials for cell cultures, targeted drug delivery, and ultrasound imaging in biological and medical research. Ebina et al.^2^ reported that oxygen and air BNBs are effective tools for growing living things. The authors experimentally showed the accelerated growth of various living things such as Brassica campestris, sweetfish, rainbow trout, and DBA1/J mice using BNBs. BNBs are also used as contrast agents. It has been established that the acoustic response of contrast agents is directly related to the NB size of the agent. Several researchers have demonstrated the potential for improving ultrasound contrast sensitivity by changing the size and size distribution of the BNBs used^10–14^. Talu et al.^10^ demonstrated that the pulse shapes of echoes from groups of uniformly sized bubbles are almost uniform, while those of polydisperse bubbles are not. They also stated that the optimization of the size distribution of contrast agents has the potential to enhance the sensitivity of contrast imaging. However, such a system could be influenced by ambient conditions and variations in the ambient conditions after injection into the incubation system. Additionally, the body may change the size distribution and concentration of the BNBs. Fully exploiting these possibilities requires a thorough understanding of the behavior of BNBs.

In this study, we investigated the coarsening behavior of CO_2_ BNBs in distilled (DI) water through correlation analysis between BNB concentration and the total volume of BNBs over time. BNBs were generated using carbon dioxide (CO_2_) gas and distilled (DI) water through a gas–liquid mixing method^15^. The presence of generated BNBs was investigated using attenuated total reflectance Fourier transform infrared (ATR-FTIR) spectroscopy, and their size distribution and concentration were studied over time using a nanoparticle tracking analysis (NTA) method. This paper demonstrates that BNBs grow over time by diffusing from smaller to larger BNBs consistent with an Ostwald ripening phenomenon.

## Experimental setup

### Materials

Carbon dioxide (CO_2_) gas (purity: 99.9%, Shinyoung Gas Co., Republic of Korea), which can be identified using infrared spectroscopy, and distilled (DI) water (No. 119, HPLC grade, Duksan Pure Chemicals Co., Republic of Korea) were used to generate BNBs in water. In addition, a 4-mL vial (SL.Vi1151, SciLab Korea Co., Ltd., Republic of Korea) and its screw cap (SL.Vi1164, SciLab Korea Co., Ltd.) were prepared by cleaning with DI water to eliminate dust from the vial, cap, and septa. All other materials were used as received.

### Generation of bulk nanobubbles

In this study, a gas–liquid mixing (agitation) method^15^ was used to generate BNBs in water using a nanobubble generator consisting of a vial holder and linear actuator. The actuator was driven by an electric motor. The rotational motion generated by the motor was converted into linear motion by a crank. The linear motion was transmitted using a connecting rod to the vial holder. Thus, the vial holder was shaken up and down to mix the CO_2_ gas and water in the vial. The following settings were used to generate the BNBs in water: a motor velocity of 90 rpm, 20 cm stroke, and a gas–liquid ratio of 1:1. Two BNB solutions with different BNB concentrations were generated: case 1 (low) at approximately 3 × 10^8^ NBs/mL and case 2 (high) at approximately 5 × 10^8^ NBs/mL.

### Nanobubble analysis

The NB analysis in water was carried out with a nanoparticle tracking analysis (NTA) method using an NTA instrument with a blue polarized 405 nm laser (NanoSight LM10-HSBFT14, Quantum Design Korea, Republic of Korea). This NB visualization technique provided size, count, and concentration measurements. Using the laser light to illuminate the NBs enabled the detection of their Brownian motion and light scattering properties. The NBs were visualized directly in the water and captured using a CCD camera to obtain the bubble size distribution, consisting of bubble diameter and concentration. The NB concentration is the total number of NBs (10–1000 nm in diameter) per milliliter. Each measurement was recorded more than five times.

### Fourier transform infrared spectroscopy

Many gases (CO_2_, CO, CH_4_) are infrared (IR) active. However, CO_2_ gas is particularly suited for IR techniques because gaseous CO_2_ and dissolved CO_2_ show different IR spectra. In this study, an attenuated total reflectance Fourier transform infrared (ATR-FTIR) spectrometer (Thermo Nicolet 6700, Scinco Co., Ltd., Republic of Korea) equipped with a liquid nitrogen-cooled mercury-cadmium-telluride (MCT) detector was used to investigate the nature of the gas in the generated BNBs. The infrared spectra were recorded at a resolution of 0.5 cm^−1^. A total of 200 scans were measured in the 650–4000 cm^−1^ range under ambient conditions.

## Results and discussion

### Generation of bulk nanobubbles

**Figure 1:**
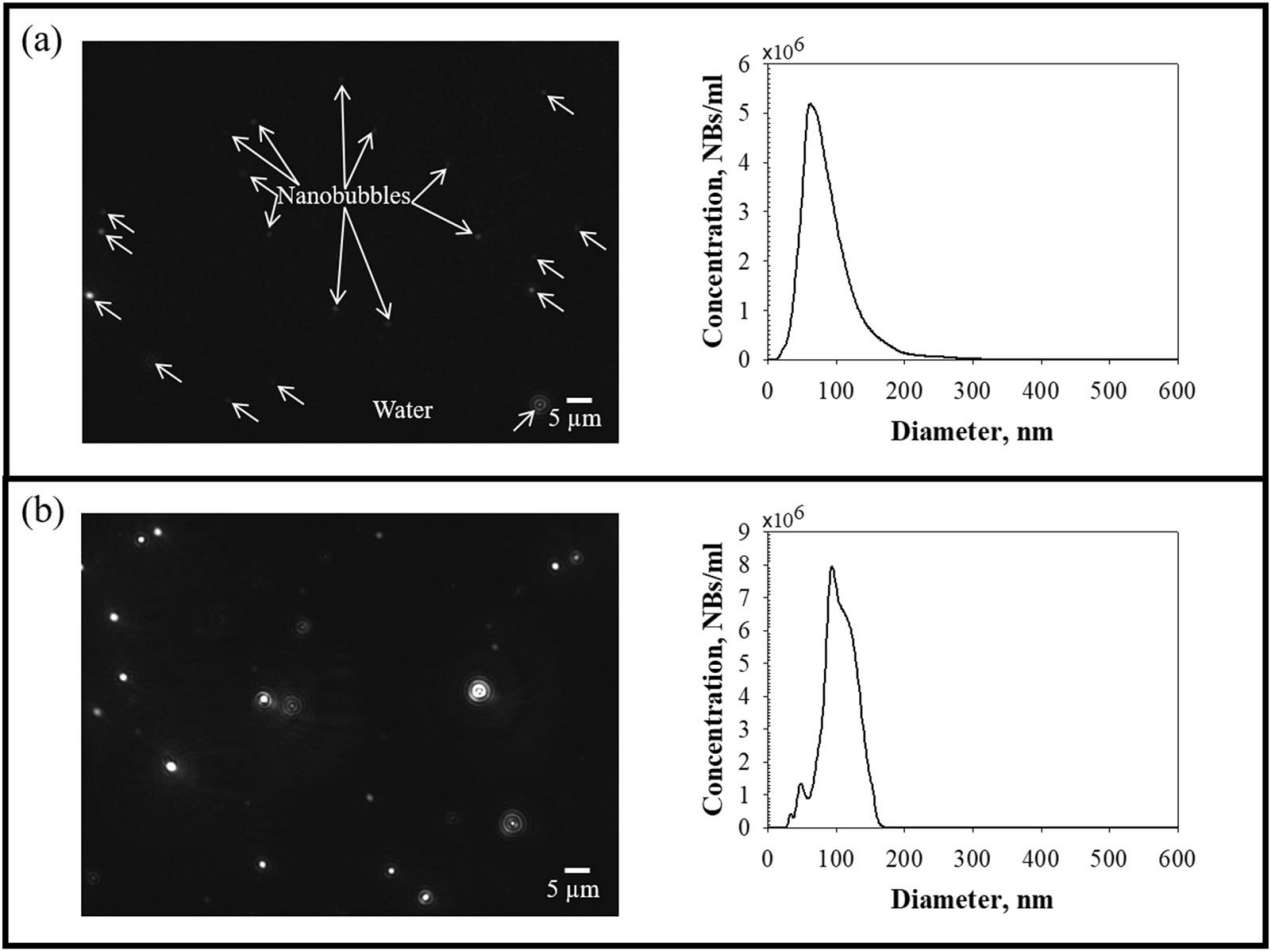
CO_2_ BNBs generated in water. **(a)** Case 1 (low BNB concentration): 3.47 ± 0.39 × 10^8^ NBs/mL, **(b)** case 2 (high BNB concentration): 4.94 ± 0.24 × 10^8^ NBs/mL. The figures on the left are images of BNBs. The black background and white dots represent the water and BNBs, respectively. The figures on the right side are the size distributions of the BNBs immediately after generation in water.

CO_2_ gas BNBs were successfully generated in DI water using a BNB generator with a linear actuator. Images of the BNBs were captured using a CCD camera immediately after generation, and size distribution was measured (Fig. 1). The laser-illuminated BNBs are represented as bright white dots in Fig. 1a,b (left). Larger BNBs were brighter than smaller ones. As shown in Fig. 1a,b (right), the size distribution of the BNBs had a single peak. The mean and mode diameters of the BNBs were 88.80 ± 9.59 nm and 70.63 ± 9.47 nm for case 1 and 109.25 ± 7.13 nm and 95.25 ± 5.31 nm for case 2, respectively. The concentrations of the BNBs were 3.47 ± 0.39 × 10^8^ NBs/mL for case 1 and 4.94 ± 0.24 × 10^8^ NBs/mL for case 2. Before BNB generation, no nano-sized particles were detected in the DI water. Based on these results, we concluded that the BNBs were successfully generated by agitating CO_2_ gas and DI water.

The existence and stability of BNBs have been discussed with regard to their high internal pressure. According to the Young–Laplace equation (Eq. 1), the pressure difference between the inside and outside of a bubble is inversely proportional to a bubble’s diameter:

1 $$P_{in} - P_{amb} = \frac{4\sigma }{d}$$

where *P*_*in*_, *P*_*amb*_, *σ*, and *d* are the internal pressure of the BNB, ambient pressure, surface tension of the liquid, and the diameter of the bubble, respectively. In this study, the internal pressure of a BNB is approximately 3.40 MPa according to this equation (using *d* = 88.50 nm and *σ* = 0.073 N/m). Therefore, BNBs are expected to be stable at ambient conditions due to the high internal pressure and should collapse quickly.

Moreover, according to Ljunggren et al.^16^, the lifetime of a bubble is described as

2 $$t = \frac{{Kd_{0}^{2} }}{12RTD},$$

where *K*, *d*_0_, *R*, *T*, and *D* are the Henry’s law constant, bubble diameter at *t* = 0, gas constant, temperature, and diffusion constant, respectively. Thus, the calculated lifetime of a BNB in this study is approximately 0.49 μs (using *K* = 2.98 × 10^3^ J/mol, *d*_0_ = 88.50 nm, *R* = 8.314 J/(K·mol), *T* = 298.15 K, and *D* = 1.60 × 10^−9^ m^2^/s).

However, recent experimental studies have shown that stable BNBs exist under atmospheric pressure, and their lifespans exceed analytic expectations^17,18^. Investigators have revealed that the BNB interface consists of hard hydrogen bonds similar to those found in ice and gas hydrates^8,19,20^. It is believed that the hard interface leads to reduced diffusivity of gas molecules in BNBs and high surface tension, helping maintain the kinetic balance of BNBs against the high internal pressure.

**Figure 2:**
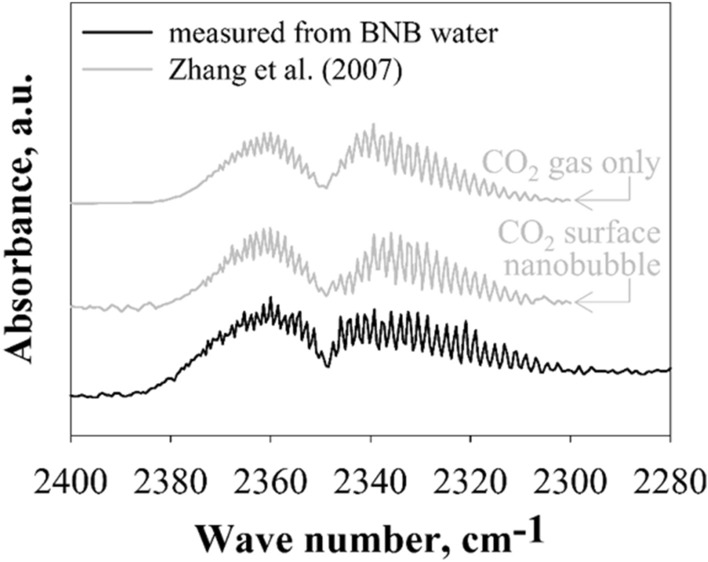
ATR-FTIR spectra of gaseous CO_2_ in surface nanobubbles and BNBs. The spectrum shows two groups of distinct fine peaks attributed to CO_2_ gas molecules. The spectrum of BNB suspensions demonstrates the formation of gaseous CO_2_ bubbles.

ATR-FTIR spectroscopy was used to detect the BNBs created from CO_2_ gas in this study, and the ATR-FTIR spectra of the generated BNBs were recorded. Figure 2 shows the ATR-FTIR spectra of gaseous CO_2_ in the BNBs. The results show two branches with fine lines at about 2300–2380 cm^−1^ (solid black line in Fig. 2). The resultant IR spectrum represents molecular absorption and transmission; the molecules absorb specific frequencies characteristic of their structures. As a result, each sample has a molecular fingerprint, and no two molecular structures create the same IR spectrum. Therefore, IR spectroscopy is useful for several types of analyses. Since gaseous and dissolved CO_2_ gas exhibit distinctly different IR spectra^21^, IR spectroscopy can directly identify the phase state of the CO_2_ molecules. Lohse et al.^21^ showed that the IR spectrum of gaseous CO_2_ consists of two branches with fine lines at 2300–2380 cm^−1^, while dissolved CO_2_ in water exhibits a single peak at about 2340 cm^−1^. Similarly, Zhang et al.^22^ demonstrated experimentally that a very thin gas domain (i.e., surface nanobubble) consists of gaseous CO_2_ with a lifespan > 1 h. These authors performed ATR-FTIR spectroscopy and showed that the ATR-FTIR spectrum measured from surface nanobubbles is the same as CO_2_ gas (solid gray lines in Fig. 2). The ATR-FTIR spectrum obtained from the BNBs in this study showed the same shape and band positions (solid black line) as a standard gaseous CO_2_ spectrum. This result reveals that the BNBs in this study were filled with CO_2_ gas.

### Coarsening of bulk nanobubbles

**Figure 3:**
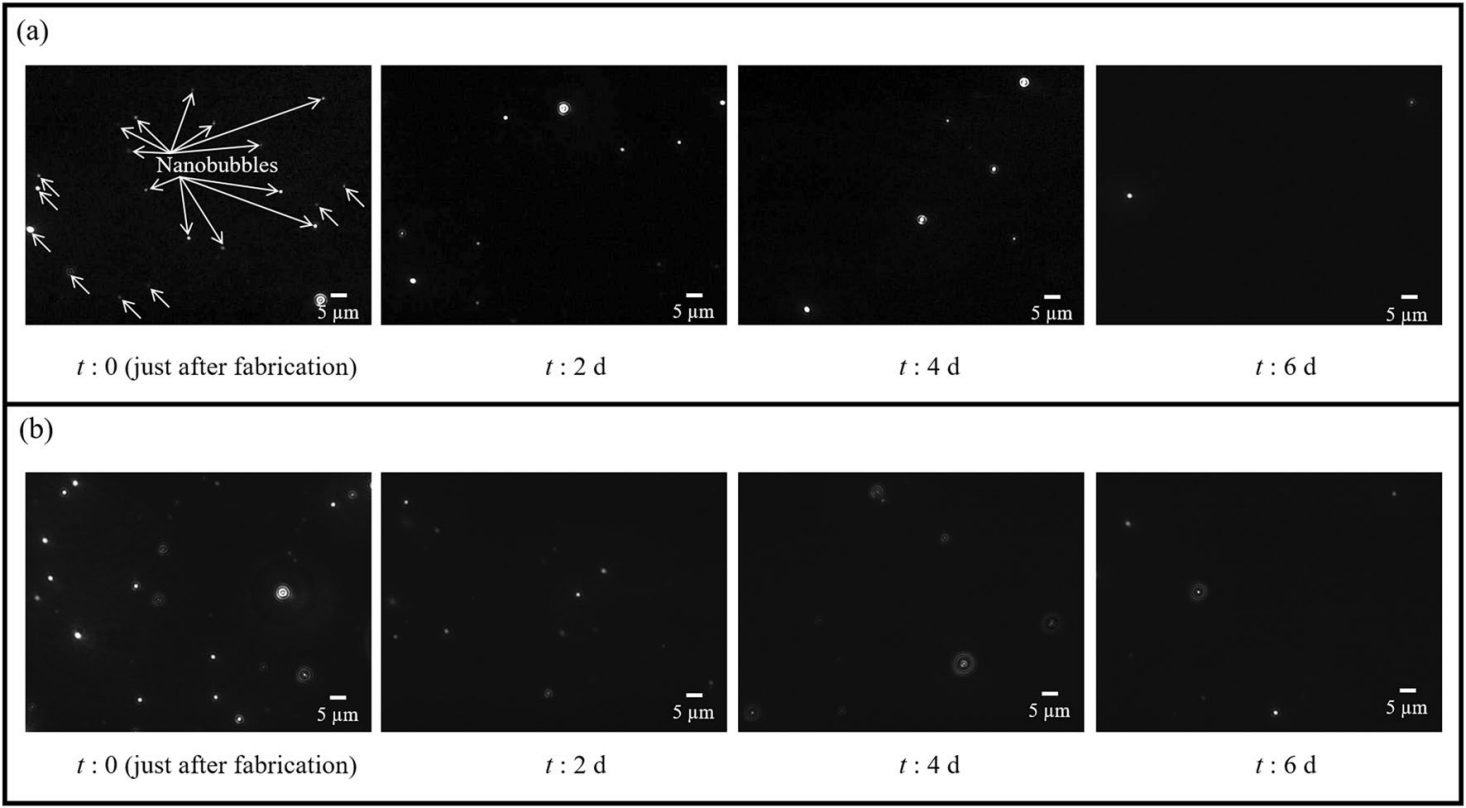
Images of BNBs in water over time. **(a)** Case 1, **(b)** case 2.

The coarsening behavior of the generated CO_2_ BNB suspensions was observed over a 6-day aging study. Images of the BNBs in water taken over time showed that the number of BNBs (bright white circles against the dark background of water in Fig. 3) decreased over time.

**Figure 4:**
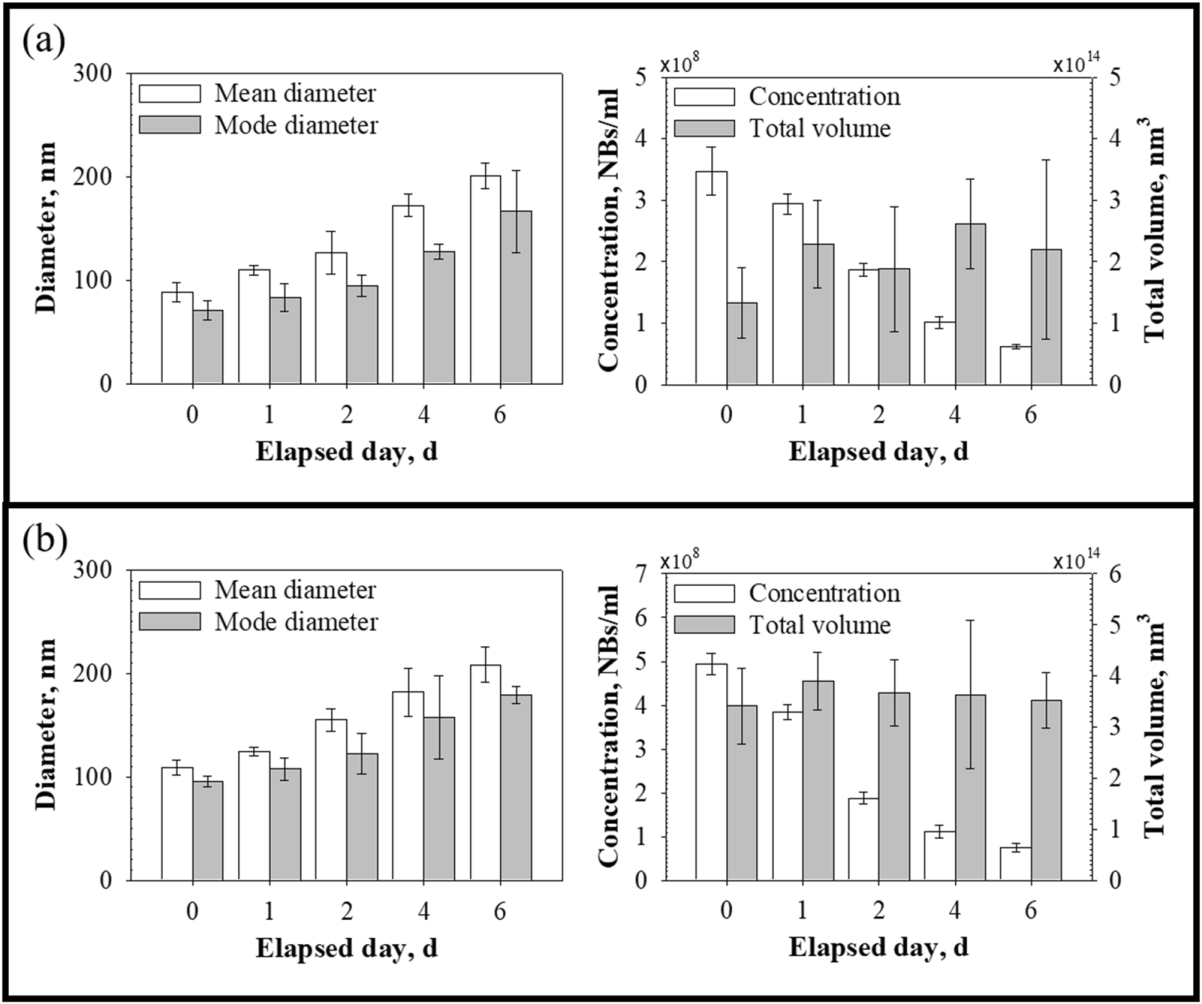
Diameters and concentration of BNBs in water. **(a)** Case 1, **(b)** case 2. The figures on the left are the mean and mode diameters, and the right side of the figures shows the concentration and total volume of BNBs.

For low BNB concentrations (case 1), the mean and mode diameters of BNBs were initially 88.80 ± 9.59 nm and 70.63 ± 9.47 nm, respectively, and the concentration of the BNBs was 3.47 ± 0.39 × 10^8^ NBs/mL (Fig. 4a). The mean and mode diameters of the BNBs increased during the aging test. In contrast, the concentration of BNBs decreased over time. After 2 days, the BNB concentration had reached half the initial concentration. After 6 days of aging, the mean and mode diameters were 201.00 ± 12.12 nm and 166.33 ± 39.70 nm, respectively, and the BNB concentration was 0.61 ± 0.03 × 10^8^ NBs/mL. In short, the BNBs showed a continuous increase in diameter. Furthermore, the measured diameters did not show a sudden and drastic increase, as seen after a coalescence event. Moreover, during the aging test, the total volume of BNBs remained almost unchanged (Fig. 4a). The time evolution of the total volume of the BNBs was evaluated by one-way analysis of variance (ANOVA). The P-value calculated, 0.08, was much larger than the significance level (0.05). This result indicates that the change in total volume was negligible over 6 days. Similarly, for high BNB concentrations (case 2), the BNB diameters increased linearly as the concentration of BNBs decreased. The changes in mean and mode diameters were from 109.25 ± 7.13 nm and 95.25 ± 5.31 nm to 208.33 ± 16.77 nm and 179.03 ± 8.54 nm, respectively. The change in concentration was from 4.94 ± 0.24 × 10^8^ to 0.74 ± 0.09 × 10^8^ NBs/mL. No change in the total volume of BNBs was observed over time (Fig. 4b). Therefore, we can infer that most of the generated BNBs did not dissolve during the aging test, and the changes in diameter and concentration were largely based on CO_2_ mass transfer between the BNBs.

The growth time for bubbles in an oversaturated solution can be calculated based on Epstein–Plesset theory^23^:

3 $$\varepsilon^{2} = 1 + x^{2} ,$$

4 $$\varepsilon = \frac{r}{{r_{0} }},$$

5 $$x^{2} = \frac{2\alpha }{{r_{0}^{2} }}t,$$

6 $$\alpha = \frac{{D\left( {c_{i} - c_{s} } \right)}}{\rho },$$

where ε is the ratio of the bubble radius to its initial radius, and x is a dimensionless variable proportional to the square root of the time; r and r_0_ are the radii of gas bubbles at time t and time 0, respectively; α is a positive constant; t is time; D, c_i_, c_s_, and ρ are the coefficient of diffusivity of the gas in the liquid, initial dissolved gas concentration, the concentration at saturation, and gas density, respectively. According to Eqs. (3–6), if the BNB suspension used in this study was slightly oversaturated with CO_2_ (i.e., c_i_/c_s_ ≈ 1, but > 1), BNBs with an initial mean diameter of 88.50 nm would grow to 201.00 nm in a very short period of time for case 1. The order of magnitude of the time required for bubble growth would be approximately −11 (using D = 1.60 × 10^−9^ m^2^/s and ρ = 1.81 kg/m^3^). Similarly, there was no difference in the time required for bubble growth in both cases. This result suggests that something at the gas–liquid interface hindered gas diffusion.

**Figure 5:**
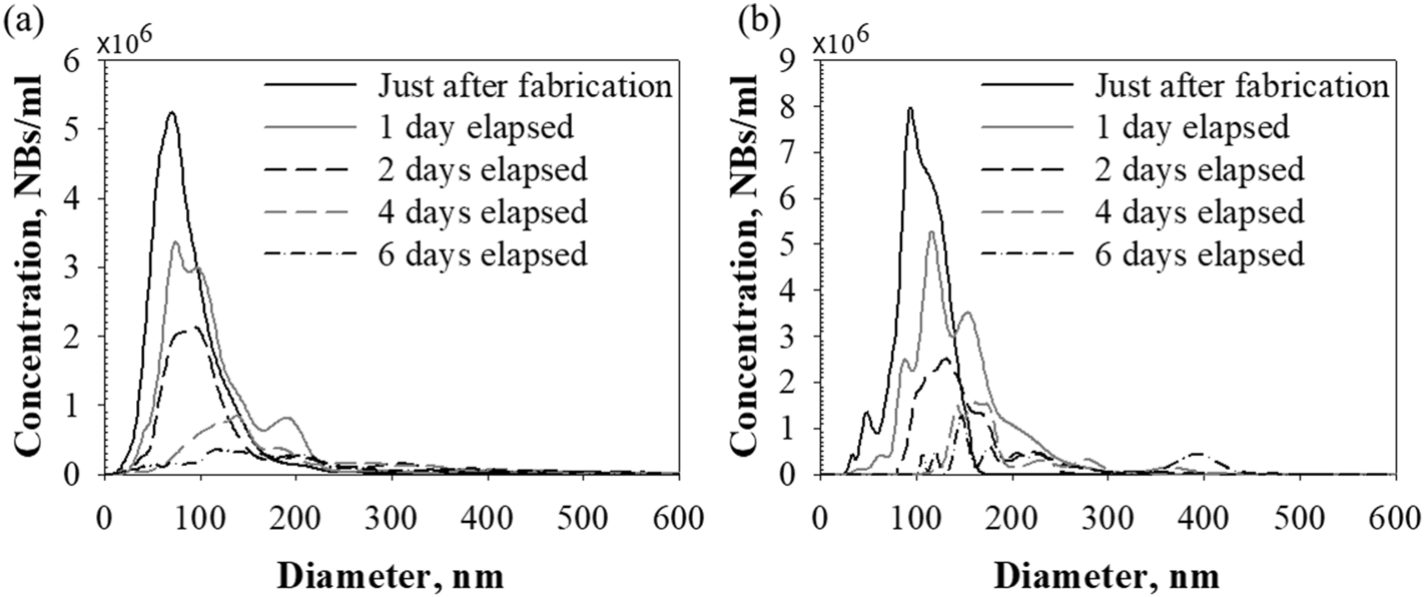
Size distribution of BNBs in water. Time evolution of size distribution: **(a)** case 1, **(b)** case 2. The initial size distribution of BNBs was in the range of 10–250 nm. The number of smaller particles decreased, while larger particles grew in size over time (approximately in the 10–530 nm range after 6 days).

The changes in the BNBs were further investigated by examining the time evolution of BNB size distribution. Figure 5 shows the time evolution of the size distribution of the BNBs in water. In each case, the BNBs initially showed a left-skewed size distribution. Few BNBs were in the > 200 nm diameter range immediately after generation. However, after 1 day, the shape of the size distribution changed to show a multi-peak distribution, and BNBs > 200 nm in diameter were apparent. In the later stages, the size distribution broadened due to the formation of larger BNBs that were not seen in the early stages. Overall, the number of BNBs decreased, and their size distribution broadened over time. Relatively large bubbles tended to appear, and relatively small bubbles tended to disappear over 6 days.

This result suggests that a spontaneous thermodynamically-driven process occurred in the CO_2_ BNB suspensions. Generally, larger particles are more energetically stable than smaller particles. As a system moves toward lower overall energy, molecules on the surface of smaller particles tend to detach and diffuse through the solution. The diffusing molecules then attach to the surface of larger particles and are absorbed into them. Therefore, the number of smaller particles decreases while larger particles grow in size^24^. A large number of relatively large BNBs were observed in the early stages of the aging test in the diameter range of 150–250 nm. Thus, gas molecules detaching from smaller BNBs may have been absorbed into these larger BNBs, further increasing their concentration. Since the size distribution peak shifted to the right over time (Fig. 5), we expect that the concentration of BNBs > 600 nm in diameter would have continued to increase if aging were allowed to continue.

Nanoscale systems have two primary particle coarsening pathways: Ostwald ripening and Smoluchowski ripening. The former is the process by which larger particles grow while smaller particles shrink. Gas molecules detaching from smaller particles diffuse through the continuous phase until they join larger nanoparticles. Moreover, this particle coarsening process can occur in microscale gas–liquid systems. Talu et al.^25^ showed that Ostwald ripening can occur even if microbubbles are initially monodispersed. In contrast, Smoluchowski ripening is caused by collisions between nanoparticles as they move through the continuous phase. Brownian motion is the random movement of particles in fluids. BNBs suspended in water also tend to move at random. These motions cause BNBs to collide, leading to the coalescence and coarsening of bubbles, thereby increasing the mean diameter of BNBs while decreasing their number. To prevent the BNBs from colliding and coalescing, BNBs would have to repel each other. It has been reported that BNBs are supported by electrically charged liquid–gas interfaces that create repulsive forces preventing BNBs from colliding and coalescing^19,26^. Additionally, when the concentration of BNBs is sufficiently low, their chance of collision becomes low^27^. Assuming the BNBs were well-dispersed in the water, the distance between neighboring BNBs would have been about 14.23 μm (about 322 times the mean radius of the BNBs) for case 1 and 12.67 μm (about 232 times the mean radius of the BNBs) for case 2 immediately after generation. Thus, collisions between the BNBs would have been infrequent in this study. Therefore, Ostwald ripening was likely the predominant particle coarsening mechanism. Moreover, the BNB diameters over time presented in Fig. 4 did not show sudden increases in BNB size corresponding to coalescence events, further supporting the Ostwald ripening mechanism.

Ostwald ripening is a direct consequence of the Kelvin effect^28,29^. The solubility of a particle increases dramatically as its size decreases, according to the Kelvin equation

7 $$S\left( r \right) = S\left( \infty \right)\exp \left( {\frac{{2\gamma V_{m} }}{rRT}} \right),$$

where *S(r)* is the solubility of the medium surrounding a particle of radius *r*, *S(∞)* is the bulk solubility, *γ* is the interfacial tension, *V*_*m*_ is the molar volume of the dispersed phase, *R* is the gas constant, and *T* is the absolute temperature. Based on the Kelvin equation, smaller particles are more soluble than larger particles. Smaller particles tend to lose their molecules, which diffuse through the continuous phase to be absorbed by larger particles. Thus, relatively small particles become smaller while larger particles grow. Consequently, the number of smaller particles decreases while the number of larger particles increases. This prediction agrees well with the tendency shown in Figs. 4 and 5.

In many studies, researchers have tried to establish a connection between the particle size distribution and the coarsening mechanism^30–34^. These studies considered the shape of the size distribution a criterion for classifying the coarsening mechanism. Granqvist et al.^32^ reported that size distributions leaning toward smaller particle sizes (i.e., left-skewed size distributions) and having a sharp cutoff above a specific size are characteristic of the Ostwald ripening process. In contrast, right-skewed size distributions (i.e., those that either lean toward larger particle sizes or are log-normal) have been attributed to Smoluchowski ripening.

In our system, although the number of BNBs tended to decrease and their size distribution broadened over time, the time evolution of the BNBs showed a right-skewed size distribution (Fig. 5). Despite the right-skewed distributions of particles, Ostwald ripening was dominant during the particle coarsening process^33–35^. Datye et al.^34^ showed that the shape of a particle size distribution cannot be used as a criterion for determining the dominant coarsening mechanism, especially when both Ostwald and Smoluchowski ripening processes can co-occur. In addition, De Smet et al.^36^ demonstrated experimentally that the droplet size distribution in a stabilized emulsion of tetradecane in water was symmetrical, significantly different from the size distribution predicted by the Lifshitz–Slyozov–Wagner (LSW) theory.

**Figure 6:**
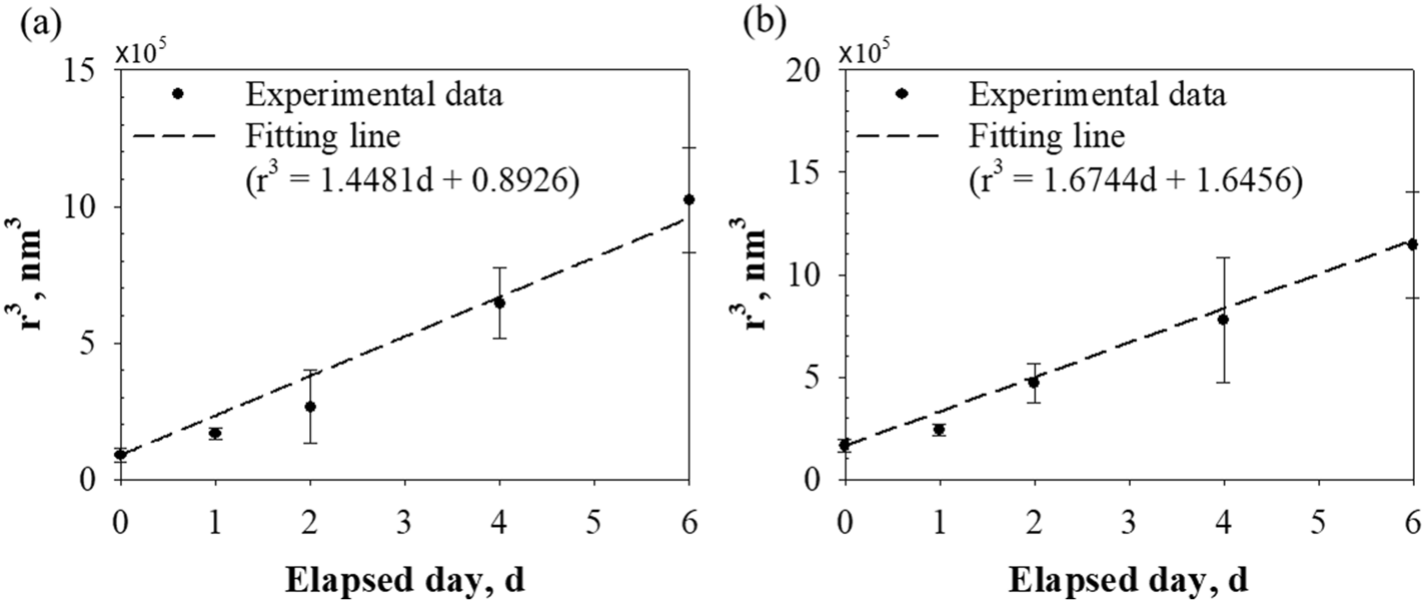
Growth rate of BNBs. **(a)** Case 1, **(b)** case 2. The measured cubic radius of NBs increased linearly with time, the same trend predicted by the LSW theory for Ostwald ripening.

Figure 6 presents the time evolution of the cubic BNB radius r^3^. In the aging test, the measured cubic radius increased linearly with time, and the slopes of the fit line were about 1.45 × 10^5^ nm^3^/day for case 1 and about 1.67 × 10^5^ nm^3^/day for case 2. In other words, the cubic radius resembled the trend predicted by the LSW theory for Ostwald ripening: the mean particle radius r was proportional to time t (i.e., r^3^ ~ t)^37,38^. This observation could be attributed to the following reasons: (1) the collision of neighboring BNBs did not occur due to the low concentration of BNBs and the negatively charged bubble surface; (2) LSW theory explicitly omits particle aggregation^39^. Therefore, these results indicate that BNBs underwent Oswald ripening. Relatively small BNBs disappeared due to dissolution, and larger BNBs grew through mass transfer between BNBs instead of coalescence.

## Conclusions

In the present study, CO_2_ BNB suspensions were prepared in water using a gas–liquid mixing method, and their particle coarsening mechanism was investigated using an NTA method. The BNBs were generated with CO_2_ gas and DI water using a BNB generator with a linear actuator. The ATR-FTIR spectra of BNBs showed two branches with fine lines at 2300–2380 cm^−1^, revealing that the BNBs were filled with CO_2_ gas. During a 6-day aging test, the BNBs showed a continuous increase in diameter. The measured diameters showed no sudden increases in BNB diameter, as would be expected after a coalescence event. Although the concentration of BNBs decreased over time, the total volume of BNBs remained unchanged. These results indicate that most of the generated BNBs did not dissolve; instead, mass transfer occurred between the BNBs. In addition, the BNBs had a left-skewed size distribution. While the size distribution of the BNBs initially showed a single peak, the size distributions after aging included two or three peaks of larger diameters. Meanwhile, the size distribution peak right-shifted over time. This result indicates that relatively small bubbles decreased in number, and relatively large bubbles increased in size over time. Moreover, the time evolution of the cubic BNB radius r^3^ increased linearly with time, and the cubic radius resembled the trend predicted by the LSW theory for Ostwald ripening (i.e., r^3^ ~ t). This observation can be attributed to the following effects: (1) the chance of BNB collision was low due to the low BNB concentration and negatively charged bubble surfaces, and (2) LSW theory explicitly omits particle aggregation. This study is a first attempt to investigate the behavior of BNBs over time in terms of mass transfer. It elucidates the mechanism of BNB coarsening in suspensions, providing valuable information for biomedical applications.
